# Community Champions for Safe, Sustainable, Traditional Food Systems

**DOI:** 10.1093/cdn/nzz119

**Published:** 2019-10-21

**Authors:** Kathleen Yung, Casey Neathway

**Affiliations:** 1 Wellness Program Services, First Nations Health Authority, Vancouver, British Columbia, Canada; 2 Environmental Public Health Services, First Nations Health Authority, Kamloops, British Columbia, Canada

**Keywords:** Indigenous populations, First Nations, nutrition, food security, food safety, public health, environmental health, food preservation, Indigenous food systems, Community Champions

## Abstract

Access to traditional Indigenous foods is a priority to improve food security and recognize the role of food in sustaining cultural and social connections. First Nations Health Authority (FNHA) is Canada's first province-wide, Indigenous-led health authority and delivers services in a community-driven manner. FNHA collaborated with First Nations to implement a Community Champion model, whereby each Nation could identify an individual who worked in food programming to attend a train-the-trainer workshop on safe food preservation methods. The Champions then took this knowledge, along with provided resources, to lead canning workshops in their home communities. Throughout the first year, a community of practice was nurtured, and a gathering of this community was held at the end of the first year. Nations were able to meet food safety considerations through interactive learning, and access to traditional Indigenous foods was strengthened. The Community Champion model supports capacity building and creates a community of practice.

## Setting the Case in Context

The First Nations Health Authority (FNHA) was established through multiple tripartite agreements, starting in 2006, between First Nations in British Columbia (BC), Canada; the Province of BC; and the Government of Canada (1). It is the only such province-wide health authority of its kind in Canada, and “is responsible for planning, management, service delivery and funding of health programs, in partnership with First Nations communities in BC” (2). FNHA seeks to deliver health services with a focus on cultural safety and humility and is guided by 7 directives, the first of which is to be “community-driven, Nation-based” (3). FNHA is responsible for delivering programs related to food safety and nutrition (4). By delivering these programs in a manner that is responsive to the direction of First Nations communities, FNHA is a partner in reducing barriers to food security and increasing access to traditional foods in a manner that reduces the risk of foodborne illness.

The importance of traditional foods for spiritual, emotional, and physical health has frequently been expressed to FNHA. Access to culturally appropriate and relevant traditional foods is a determinant of Indigenous health and well-being, has been shown to strengthen social and health supports, and has been incorporated into Indigenous youth suicide prevention plans (5). Traditional foods not only provide connections to culture, community, and the land, but are also a more nutritionally suitable food choice, and their consumption leads to higher daily intakes of vitamins, minerals, and protein (6). In BC, on days where traditional foods are consumed, there were significant improvements noted in diet, including an increase in the consumption of protein, vitamin D, iron, zinc, and potassium (7).

This study will describe how FNHA supported increasing access to the processing and sharing of safely preserved traditional foods through the facilitation of a Community Champion model and the development of accompanying resource materials. Engaging Community Champions recognized the positive social impacts of sharing foods and traditional food systems, including access to nutrient-rich harvested foods, while the curriculum development and engagement of environmental health professionals ensured advice given would lead to decreased risks of foodborne illness.

## Information on Participants

FNHA provides services to 203 First Nations across the province of BC. To ensure services are delivered in a manner that is culturally appropriate, community-driven, and Nation-based, FNHA operates within a regional structure: the boundaries of each of the 5 regions align with those of the province's regional health authorities, to allow collaboration and consistency in the provision of care. Individuals making up a representative sample from each of the 5 regions participated in the pilot project, with the intention that their knowledge would be shared within their regions, between neighboring communities, and within their own First Nations.

## Nature of the Study

Community Champions were identified through existing relationships that FNHA staff had in Nations and through conversations about further supporting and advancing their work. Community Champions were chosen from each of the 5 FNHA regions to ensure an equitable geographic representation. Priority was given to participants from Nations who had not already had a Champion attend a workshop and who had Food Champions that were already supporting nutrition programming in the community.

Champions attended train-the-trainer workshops on food preservation and canning, hosted by the Greater Vancouver Food Bank Society and sponsored by FNHA (8). Three sessions were held over 1 y and a waiting list was maintained for Champions to attend future workshops. The sessions included hands-on training in both hot water bath and pressure canning, workshop facilitation and adult education training approaches, and relationship building between participants. Semistructured focus group discussions were conducted at each workshop with questions to participants that included:

- What supports (skills, supports) would you need that could assist your community to improve access to traditional and seasonal foods?
- Who are the key people and what are the linkages in your community that can support getting more traditional foods into various settings (e.g. schools, childcare facilities, care homes)?
- Are there external partners that can support getting more traditional and seasonal foods into the above settings?

The information gathered informed next steps to support Community Champions and priority areas for further capacity building. Key requests from the Community Champions included:

- A gathering for peer-to-peer mentoring, cross-regional sharing of best practices, food programming initiatives, improve food trading opportunities, and more technical food safety workshops; and
- Empowerment through skills and capacity building:
  - Integrating mental wellness and food approaches;
  - Facilitation, listening, and public speaking skills; and
  - Planning and implementation of canning/food programs and looking at holistic food program approaches (with schools, men's groups, therapy groups, food harvesters).

Participants in the workshop formed a community of practice and developed relationships and common understandings of opportunities for future work.

FNHA purchased ∼200 All-American brand pressure canners, as well as jars and lids, for distribution to First Nations. These resources were provided to each Community Champion upon completion of the train-the-trainer workshop, and on request to other First Nations who did not attend the workshops. FNHA would also proactively offer a canner and technical support if needed to any Nation doing community-level canning work, with the intention of increasing resource capacity and reducing barriers to safe food preservation.

**FIGURE 1:**
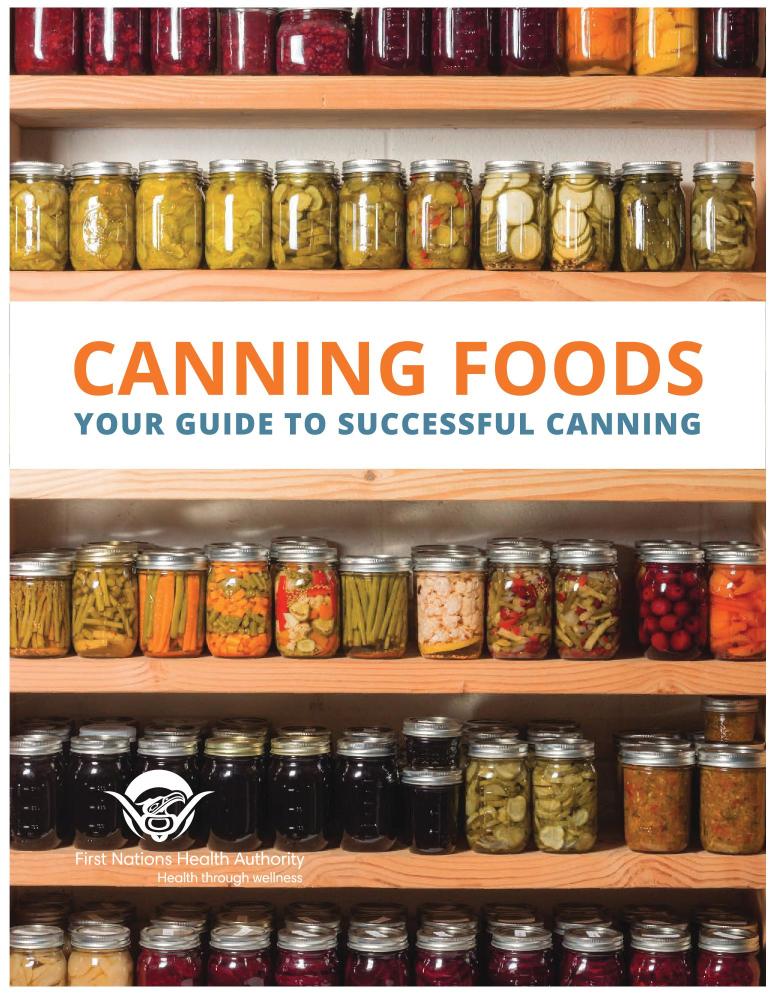
Canning resource guide cover page.

At the end of the first year of the project a gathering was held to further Champion education and discuss successes and challenges. The gathering included hands-on canning work (both pressure and hot water bath); facilitation and public speaking training; program planning and grant writing workshops; and food safety education. FNHA developed a resource guide (Figure 1) to support Community Champions that was relevant to First Nations in BC, with the proper recognition of local foods, climates, and environments. Technical information from existing resources was referenced, with local considerations added where appropriate. The guide only included recipes that had been properly vetted by a process authority to ensure food safety, and processes included in the guide were a low risk of causing foodborne illness if followed precisely. Future iterations of the guide will include community-submitted recipes, as FNHA develops relationships with appropriate process authorities to ensure their safety.

To further ensure the guide was relevant to First Nations, photographs and quotes from community members were used throughout. FNHA Communications staff were responsible for the design, editing, layout, and compilation of the guide, and Environmental Health and Wellness Program staff developed the raw material and technical details. Select Community Champions were given the opportunity to review and provide feedback on the guide before its finalization. An initial print run of 750 copies was distributed to First Nations at community gatherings, through Champions, and on request. The FNHA website hosts a digital (portable document format; PDF) copy of the guide for those who were unable to access a physical copy (9).

## Findings

By providing training, resources, and support to Champions to deliver food preservation programs in their own communities, barriers to accessing safe traditional foods were reduced, and an increased desire to serve these foods was seen in communities.

The development of a community of practice, including more than 30 Champions and FNHA staff (Table 1), allowed peer-to-peer exchange of technical knowledge, as well as program development and relationship building. The Champions, by attending canning events in neighboring communities, contributed to the organic growth of the community of practice and continued improvement in access to traditional foods. The community of practice connects via social media on an ongoing basis to share their stories and best practices.

The publication and advertising of the resource guide raised the profile of safe canning for First Nations members who may not have been formally involved in food preservation. The very high demand for printed copies of the guide indicates community members are interested in safely preserving traditional foods.

## Lessons Learned

The preservation of seasonal (i.e. contemporary) and traditional foods is pivotal in strengthening social connections, improving food security and nutrition (especially during the winter months), and increasing resilience if built into emergency response planning (10). Establishing a network of Champions across BC can support First Nations to share best practices, program initiatives and lessons learned, and methods of successful community mobilization and support.

To improve access to traditional foods in many regulated facilities in BC (e.g. public restaurants or care facilities), governmental support will be required. The provincial Ministry of Health has jurisdiction over food safety legislation, and although this legislation does not necessarily have force in First Nations communities, it restricts access to traditional foods in provincially regulated facilities. Modifications to this legislation, or of its application, are necessary to allow better access to game meats, gathered foods, and traditionally caught fish.

An unexpected outcome that resulted from this project was the interest of the Community Champions to build food preservation and food security strategies into their emergency response planning. Many First Nations in BC have been heavily impacted by recent climate change activities (11), including flooding and wildfires. These realities highlight the importance of access to traditional foods and ensuring resources are secured in times where store-bought foods are disrupted and not readily available.

**TABLE 1:** Numerical case details

# of Community Champions	32
# of pressure canners purchased	200
# of train-the-trainer workshops	3
# of canning guides printed	750

## Notes

Funding for the program was provided by the First Nations Health Authority.

Author disclosures: CN and KY, no conflicts of interest.

This article appears as part of the supplement “Proceedings of the Third Annual Conference on Native American Nutrition,” sponsored by the Shakopee Mdewakanton Sioux Community's Seeds of Native Health campaign through a gift to the University of Minnesota, USA. The guest editors of the supplement, Treena Delormier and Mindy S Kurzer, have no conflicts of interest. The opinions expressed in this publication are those of the authors and are not attributable to the sponsors or the publisher, Editor, or Editorial Board of *Current Developments in Nutrition*. Publication costs for this supplement were defrayed in part by the payment of page charges.

Abbreviations used: FNHA, First Nations Health Authority; BC, British Columbia.
